# Selectivity Studies and Free Energy Calculations of AKT Inhibitors

**DOI:** 10.3390/molecules29061233

**Published:** 2024-03-10

**Authors:** Haizhen A. Zhong, David T. Goodwin

**Affiliations:** Department of Chemistry, University of Nebraska at Omaha, Omaha, NE 68182, USA; dtgoodwin@unomaha.edu

**Keywords:** AKT, covalent docking, selectivity, anticancer, drug design

## Abstract

Protein kinase B (PKB) or AKT protein is an important target for cancer treatment. Significant advances have been made in developing ATP-competitive inhibitors and allosteric binders targeting AKT1. However, adverse effects or toxicities have been found, and the cutaneous toxicity was found to be linked to the inhibition of AKT2. Thus, selective inhibition of AKT inhibitors is of significance. Our work, using the Schrödinger Covalent Dock (CovDock) program and the Movable Type (MT)-based free energy calculation (ΔG), yielded small mean errors for the experimentally derived binding free energy (ΔG). The docking data suggested that AKT1 binding may require residues Asn54, Trp80, Tyr272, Asp274, and Asp292, whereas AKT2 binding would expect residues Phe163 and Glu279, and AKT3 binding would favor residues Glu17, Trp79, Phe306, and Glu295. These findings may help guide AKT1-selective or AKT3-selective molecular design while sparing the inhibition of AKT2 to minimize the cutaneous toxicity.

## 1. Introduction

Protein kinase B or AKT is a serine/threonine kinase essential in the phosphoinositide 3-kinase (PI3K)/AKT/mammalian target of the rapamycin (mTOR) pathway. Dysregulation, mutations, and malfunction of this pathway can induce various types of cancer, such as B cell malignancies, breast cancer, and renal cell carcinoma [1,2,3], and diabetes [4]. There are three known subtypes of AKT proteins, AKT1 (PKBα), AKT2 (PKBβ), and AKT3 (PKBγ). AKT1 is linked to cell survival, growth, and antiapoptotic behavior [5]; AKT2 deletions lead to a type 2 diabetic phenotype with impaired glucose uptake [6]; and the absence of AKT3 is related to neuronal malfunctions and neuroinflammation [7]. Further studies show that AKT2 is related to breast cancer metastasis, and AKT1 plays an important role in the local cancer initiation process [8,9]. There are three domains on the AKT, a pleckstrin homology (PH) domain in the N-terminus, the central catalytic kinase domain, and the C-terminal regulatory domain. Upon activation, PIP3 binds to the PH domain, causing conformational changes and exposing Thr309 of AKT1 for phosphorylation of the kinase domain, followed by a secondary phosphorylation of AKT1 at the C-terminal Ser473, which is mediated by the mTOR complex 2 (mTORC2). The respective phosphorylation sites of AKT2 and AKT3 are on AKT2 (Thr309/Ser474) and AKT3 (Thr305 and Ser472) [10,11,12].

Mutations in AKT1-3 occur in 3–5% of human cancers, and research has shown that most mutations such as AKT1 E17K, L52R, and Q79K have no effect on AKT inhibitors. However, mutants AKT1 D323H and AKT2 W80C cause resistance to the allosteric AKT inhibitor MK-2206 but confer no effect on ATP-competitive inhibitors [13].

**Figure 1:**
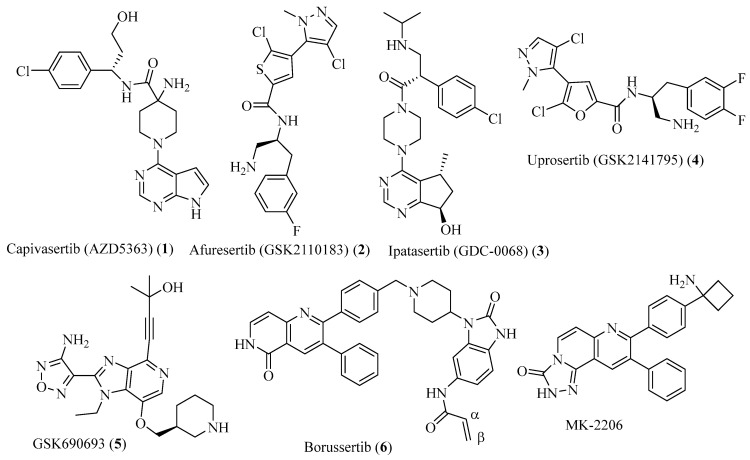
AKT inhibitors that are under clinical trials.

Given the importance of AKT in the PI3K/AKT/mTOR pathway in regulating cell proliferation, the discovery of AKT inhibitors has been under extensive research. Current AKT inhibitors can be classified as either active site binding ATP-competitive inhibitors, such as capivasertib (AZD5363, Figure 1, **1**) [14], afuresertib (GSK2110183, **2**) [15], ipatasertib (GDC-0068, **3**) [16], uprosertib (GSK2141795, **4**) [17], and GSK690693 (**5**) [18], or the allosteric binders, such as borussertib (**6**) [19,20] and MK-2206 [21] (Figure 1).

Compounds **1**–**6** in Figure 1 are in various stages of clinical trials. Studies show that 40.8% of patients with hormone receptor-positive and human epidermal growth factor receptor 2 (HER2)-negative advanced breast cancer had AKT pathway alterations, and the most observed adverse events with capivasertib treatment were rash (in 12.1% of patients) and diarrhea (in 9.3% of patients), and phase I/II studies showed efficacy of capivasertib as a single agent or as a combination therapy with paclitaxel and fulvestrant in hormone receptor (HR)-positive, HER2-negative breast cancer [22]. Compounds **2**–**5** are all reversible, ATP-competitive pan-AKT inhibitors, targeting the ATP-binding domain of AKT. Rash is the most observed toxicity of AKT inhibitors. AKT2 inhibition was considered to be the cause of cutaneous toxicity (rash), and thus, selective inhibition of AKT1 and sparing AKT2 activity can develop a molecule with reduced occurrence of rash [23].

In addition to the side effect of rash for the pan-AKT inhibitors, the selectivity of ATP-binding inhibitors could be an issue due to the highly conserved nature of the ATP-binding domain among kinases. Thus, it is commonly observed that reduced doses sometimes are needed to minimize the side effects, leading to poor efficacy [24].

An alternative approach to targeting both the pleckstrin homology (PH) domain and the catalytic domain has led to the discovery of AKT allosteric binders such as borussertib (**6**) and MK-2206. The α,β-unsaturated amide bond on the borussertib (Figure 1, **6**) allowed it to form covalent bonds with Cys296 and Cys310 of AKT1 (IC_50_ of borussertib for WT AKT1, 0.2 nM, for mutant C296S and C310S, 58 nM, respectively). The significantly reduced activity in the AKT1 C296S and C310S mutant confirmed the significance of Cys296 and Cys310 of AKT1 in covalent ligand binding [19].

Thus, it is critical to design AKT1-selective inhibitors to minimize the side effect of rash due to AKT2 inhibition. It is also important to design allosteric inhibitors to overcome drug resistance and improve selectivity. To help elucidate residues that are important for subtype binding and to help selective ligand design, we built thirty AKT inhibitors with inhibitory activities against AKT1, AKT2, and AKT3 and docked these thirty compounds to AKT1, AKT2, and AKT3. To identify binding residues, we enumerated all binding residues. The most frequently observed residues are important for selective ligand binding. In addition, we employed a Movable Type (MT)-based free energy (ΔG) calculation for protein–ligand interactions. The details of the MT-based ΔG calculation will be given in the method and the discussion sections.

## 2. Results and Discussion

### 2.1. Multiple Sequence Alignment

To determine the difference in binding residues among different AKT subtype sequences, we performed multiple sequence alignment on protein sequences of an AKT1 (6S9W) [25], AKT2 (3D0E) [26], and AKT3 homology model (HM, AAD24196.1) [27] using Clustal Omega [28]. The alignment of sequences is listed in the supporting information (Supplemental Information, Figure S1). Residues that are important for ligand bindings are given in Table 1. In this paper, we built two model proteins for each subtype: AKT1: 6S9W and 6S9W_L (with missing loop residues being built in); AKT2: 3D0E and 3D0E_N (with the N-terminal PH domain being attached to 3D0E); and AKT3 (GenBank ID: AAD24196.1): HM model (built from the homology modeling method) and the AF model (structure downloaded from the AlphaFold database). Please refer to the method section for details. For multiple sequence alignment, we used four sequences: 6S9W for AKT1; AAD24196.1 (GenBank ID) for AKT3; and 3D0E and AKT2_Comb (which was built by combining the N-terminal PH domain with the 3D0E sequence).

Figure S1 and Table 1 show that the binding residues are quite conserved across three subtypes of AKT. This may help explain why the molecules **1**–**5** are pan-AKT inhibitors and thus lack selectivity. The difference lies in whether the residues of a particular subtype are involved in ligand binding or not. Thus, it is this subtle difference that can be utilized to assist in selective molecular design. Based on the sequence alignment, AKT1 shares 88.7% of sequence identity with AKT2 (3D0E without the N-terminal PH domain) and 83.3% between the AKT1 and AKT2 with the N-terminal PH domain. The sequence identity between AKT3 and AKT2 is 78.8–83.3% (Figure S1).

### 2.2. Building Model Proteins

The crystal structures of AKT1 and AKT2 are readily available from the Protein Data Bank. Protein 6S9W was used as the AKT1 model protein, and 3D0E was used as the AKT2 model protein, and both were downloaded from the Protein Data Bank (www.rcsb.org) (accessed on 3 June 2023). The preparation of model proteins (6S9W and 3D0E) was described in the method section. There was a big loop with missing coordinates in 6S9W, which is far from the bound ligands with a distance of 19.15 Å between the ligand and residue Lys112. Due to the far distance, the missing loop would have minimal effect on ligand binding and thus 6S9W was used without fixing the missing residues in this big loop. However, to evaluate whether the missing loop would have a positive effect on the ligand binding, we built a 6S9W_L model with the large missing loop of Gly113 and Pro141 being built using the de novo program in the MOE [29]. Similarly, 3D0E has no coordinates for residues in the N-terminal PH domain. To evaluate the effect of the N-terminal PH domain on ligand binding, we also built a model protein for AKT2, named 3D0E_N by adding the PH domain residues back to the model protein 3D0E. There was no crystal structure available for the AKT3 ATP-binding domain. Thus, a homology model was built based on the AKT3 sequence (AAD24196.1) using the homology model in the MOE program, as described in the method section [29], using 6S9W as a template. The selection of 6S9W as a template protein to build the AKT3 model was due to the high sequence identification between AKT1 and AKT3 (82.3%, Figure S1). We also downloaded the full-length structure of the AKT3 from the AlphaFold database (https://alphafold.ebi.ac.uk/entry/Q9Y243) (assessed on 7 January 2024) [30,31] to compare the performance between the homology model (HM model) and the AlphaFold structure (AF model).

The homology modeling-derived model protein AKT3_HM and the AlphaFold model protein (AKT3_AF) were evaluated for structural quality by using the ERRAT and ProCheck programs at the UCLA SAVES v6.0 program (https://saves.mbi.ucla.edu/) (assessed on 7 January 2024) [32]. The ERRAT program reported the overall quality factor for the AKT3_HM and AKT3_AF as 89.07 and 87.85, respectively. The ProCheck program reported whether there is a violation of main chain torsional angles in terms of the Ramachandran plot. The number of residues in the disallowed regions was 1.3% for the AKT3_HM model and 1.2% for the AKT3_AF model, respectively (Supplemental Information, Figure S2). Both the ERRAT quality score and the Ramachandran plots suggested that both the HM and the AF model were structurally sound.

### 2.3. Docking Scores and Validation

**Figure 2:**
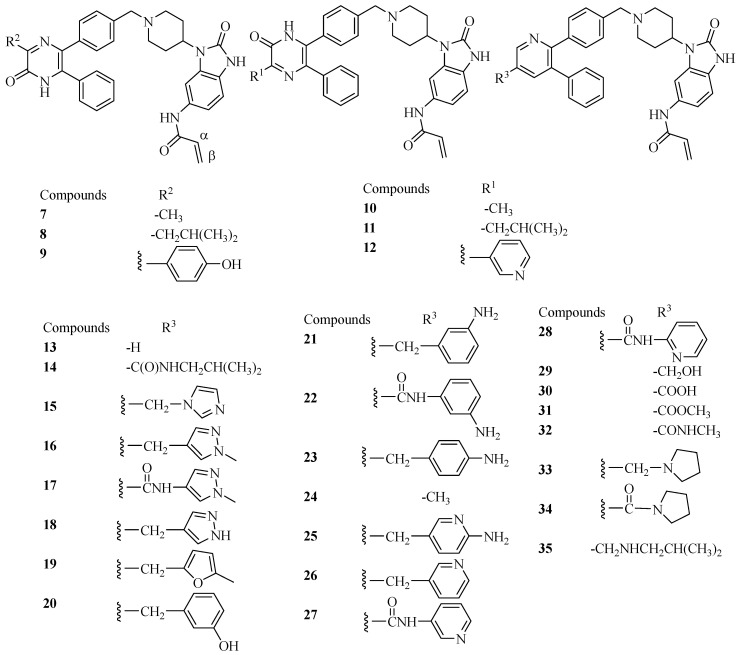
Structures of AKT inhibitors used in the docking studies.

To identify amino acids that are essential for ligand binding, we performed docking studies for the thirty AKT inhibitors (compound **6** in Figure 1 plus twenty-nine ligands in Figure 2) with inhibitory activities (IC_50_) identified for the AKT1, AKT2, and/or AKT3, respectively. The ligands were taken from the published papers [25,33]. The structures of the ligands that were used for docking studies are given in Figure 2. Please note that for the thirty compounds used for docking studies, the docking program returned all thirty docked poses except one ligand in the 6S9W_L model with docked poses not identified (Table S1). However, when we evaluated the docking scores, only those compounds with reported IC_50_ or K_i_ were included in the discussion and those with experimental IC_50_ > 20,000 nM were excluded from the calculations of ΔΔGs. Based on the reported IC_50_ values, this resulted in thirty compounds for AKT1, twenty-eight for AKT2, and twenty-three for AKT3.

#### 2.3.1. Validation of Method

The binding affinity of the AKT/ligand complexes from the covalent docking was expressed in terms of the CovDock scores. During the covalent docking, five protein/ligand complexes were kept and the most potent (or the most negative) docking score was used as the CovDock score. To validate docking methods, we compared the CovDock scores to the free energy of binding (ΔG), which was estimated from the experimentally available IC_50_ using the following equation.

(1) $$\Delta G_{\exp}\;(\mathrm{kcal}/\mathrm{mol})=\mathrm{RT}\;\ln\;\left({\mathrm{IC}}_{50}\;\left(\mathrm{nM}\right)\times 10^{-9}\right),$$

where R = 1.987 cal·K^−1^·mol^−1^ and T = 298.15 K.

In addition to the CovDock affinity scores, we also calculated the free energy of binding (ΔG) using the Movable Type (MT)-based method and compared the MT-based ΔG to the experimentally derived ΔG as estimated using the above equation.

The Schrödinger Covalent Dock program generated CovDock scores for the AKT1 6S9W model (which contained a large gap) and the 6S9W_L model (which inserted back the missing coordinates for the big loop residues), and the MT-based free energies for thirty AKT1 inhibitors were listed in Table S1 in the Supplemental Information. Table S1 shows that the mean errors (ΔΔGs) for the CovDock method for the 6S9W and 6S9W_L models were 3.56 and 4.56 kcal/mol, respectively, versus the mean error (ΔΔG) for the MT-based method of 5.89 kcal/mol (Table S1). Both numbers were above 2.0 kcal/mol, the common threshold for a good estimation. For the same CovDock method, the 6S9W_L model yielded a larger mean error. This was no surprise to us at all because the 6S9W_L model contained an insertion of a large loop for the missing residues between Gln113 and Pro141. The minimization of this new loop would cause conformational changes to the binding pocket and thus would affect the overall ligand binding. Thus, our initial assumption of ignoring this big gap was appropriate in that this large gap was far from the ligand binding area: the distance between the ligand and residue Lys112 was 19.15 Å.

At first glance, in the case of AKT1, it appears that both the Schrödinger covalent CovDock program and the MT-based ΔG estimation yielded large errors. However, a detailed kinetic analysis using the K_i_ (nM) rather than the IC_50_ (nM) showed that the mean errors (ΔΔGs) for these three models became 2.18, 3.85, and 3.87 kcal/mol, respectively (Table 2), which showed an improvement in terms of having smaller mean errors. Please note only a few compounds were reported with K_i_ activities [25,33]. That is why the number of compounds with K_i_ activities was less than those reported in IC_50_.

On the other hand, the mean errors for the AKT2 models were much smaller than those of the AKT1 models. For the AKT2 model protein 3D0E, the X-ray structure of 3D0E started from Lys146 and missed the N-terminal pleckstrin homology (PH) domain. We built two model proteins for the AKT2: one was 3D0E without the PH domain, the other with the addition of the N-terminal PH domain of AKT2 (PDB id: 1P6S) [34] to 3D0E, which was called 3D0E_N. Please refer to the method section regarding the building of this 3D0E_N model.

The mean errors (ΔΔGs) for the 3D0E and 3D0E_N models from the CovDock and the ΔΔGs for the MT method for these two model proteins were 1.03, 1.17, 2.36, and 2.38 kcal/mol, respectively (Table 3). The close ΔΔG between the 3D0E and 3D0E_N models suggested that it was the catalytic-binding domain, not the PH domain, that made the most contributions to the AKT2 ligand binding.

The absolute mean error from the CovDock method for the AKT2 models was very impressive, with ~1.0 kcal/mol. The absolute mean error from the MT-based method, on the other hand, was slightly above 2.0 for both the 3D0E and 3D0E_N models.

Similarly, when the K_i_ (nM) rather than the IC_50_ (nM) was used, the mean errors (ΔΔGs) for the MT-based method were below 2.0 kcal/mol, at 1.12 and 0.95 kcal/mol versus 1.73 and 1.88 kcal/mol mean errors (ΔΔGs) for the CovDock method for the 3D0E and 3D0E_N models (Table 4). This indicates that in the AKT2 models, if the K_i_ values were used, the estimation of the free energy of binding by the MT method or CovDock program was accurate in predicting experimentally measured ΔG. Thus, it is reliable to use the CovDock program to investigate the protein/ligand interactions.

The Movable Type (MT)-based free energy calculation method, pioneered by Zheng and Merz [35,36], is a fast approach to calculating the binding free energy of a protein–ligand complex. Once the coordinates of a protein–ligand complex are provided, it first identifies all the atom pairs in the system and then samples over all the configurational space of the protein active site. The Helmholtz free energy is used to approximate the gas-phase Gibbs binding free energy (ΔG), and the partition functions are integrals over all possible coordinates of the protein–ligand complex, the protein, and the ligand. In a similar manner, the solvation free energy is also calculated using the Helmholtz free energy. The MT method breaks down the protein–ligand complex to the atom pair level, which simplifies the calculations on an otherwise 3D problem and thus speeds up the calculation of the ΔG. This pairwise treatment of the protein–ligand complex allows for the sampling of all configurational space in one shot without using the more time-demanding molecular dynamics simulations [37,38]. The binding free energy calculated by the MT method thus contains the contributions from both the enthalpy and the entropy. Table 2 and Table 4 showed that when the K_i_ values were used, the MT-based approach yielded reasonable estimation of ΔGs comparable to those obtained from the Schrödinger CovDock program but with a fraction of time. For the same desktop computers, the calculation of the MT-based free energy normally takes around 5 s, but it took about 25 min when using two nodes to calculate the docking scores from the CovDock program. It is true that during the docking process, the CovDock, like the Schrödinger’s Glide Dock program, not only calculates the binding affinity but also identifies the binding conformations by sampling the binding pocket. It is reasonable to presume that most of the time for the CovDock was spent on locating proper conformations for the ligand to bind. Nevertheless, the brief time used for the MT method to afford a reasonable estimation of the free energy of binding (ΔG) was still quite impressive, and we attributed this to the pairwise method adopted in the MT program. We have used the MT method to successfully predict the binding free energy of various protein–ligand interactions, including systems with mutations and covalent bonds [39,40,41]. It is true that people can use the nmode program in the AMBER software to calculate the entropy contributions and use the molecular dynamics (MDs) simulation-based Generalized Born Surface Area (GBSA) method to estimate the ΔG. However, this method requires a lengthy MD simulation [42]. Compared to the Schrödinger Covalent Dock (CovDock) method [43], the MT-based method is faster too.

#### 2.3.2. Prediction of the AKT3 Ligand Binding

Since there was no reported human AKT3 crystal structure available for the ATP-binding domain, we built a homology model of the human AKT3 protein (HM model) using the protein primary sequence from the NCBI with sequence ID AAD24196.1 and the human AKT1 (6S9W) structure as a template. The sequence identity between AKT1 (6S9W) and AKT3 (HM, AAD24196.1) was 82.3%. This high level of sequence identity supports the assumption of homology between these two structures. After the homology model was built and minimized, we docked thirty AKT inhibitors to the AKT3 HM model. The CovDock returned thirty docked poses for all ligands. However, when we reported the docking affinity, only those with specified IC_50_ values were included for accurate comparison, i.e., those entries with >20,000 nM were excluded due to the uncertainty of the IC_50_ [25,33].

In addition, we downloaded an AKT3 structure from the AlphaFold website (https://alphafold.ebi.ac.uk/entry/Q9Y243) [30,31] (accessed on 7 January 2024) and called this model protein AKT3_AF. AKT3_AF was an apoprotein and thus was structurally aligned to the 6S9W, and the borussertib of 6S9W was adopted to AKT3_AF as a binding ligand for docking studies. This AKT3_AF was prepared using the same procedures and settings as the AKT3_HM.

The absolute mean errors (ΔΔGs) predicted by the CovDock for the HM and AF models were 2.69 and 1.07 kcal/mol, respectively (Table 5). The AlphaFold-generated AF model was better than the HM model in terms of reproducing the experimentally derived ΔG. Thus, we used the coordinates obtained from the AF model to estimate the MT-based ΔG. The MT method yielded a ΔΔG of 2.22 kcal/mol, close to the experimental values. Like those of AKT1 and AKT2 models, when the K_i_ values were used, the absolute mean errors (ΔΔGs) for the HM model and for the MT-based method became lower, at 0.49 and 0.54 kcal/mol (Table 6).

The small absolute mean errors (ΔΔGs) in the AKT2 and AKT3 models and the acceptable ΔΔG of AKT1 in terms of K_i_s suggested the validity of the CovDock and the MT method, and thus, we will use the CovDock generated docked poses to discuss the protein–ligand binding interactions.

#### 2.3.3. Binding Interaction of AKT Inhibitors

As the validity of the Schrödinger covalent docking (CovDock) was confirmed, the protein–ligand interactions for the docked poses could then be identified. There are two main domains for the AKT proteins: for AKT1 and AKT2, the PH domain with residues ranging from residue 4 to 111 (for the AKT3, the PH domain consisted of residues 4 to 110) and the catalytic domain for residues between 124 and 479 (Supplemental Information, Figure S3).

**Figure 3:**
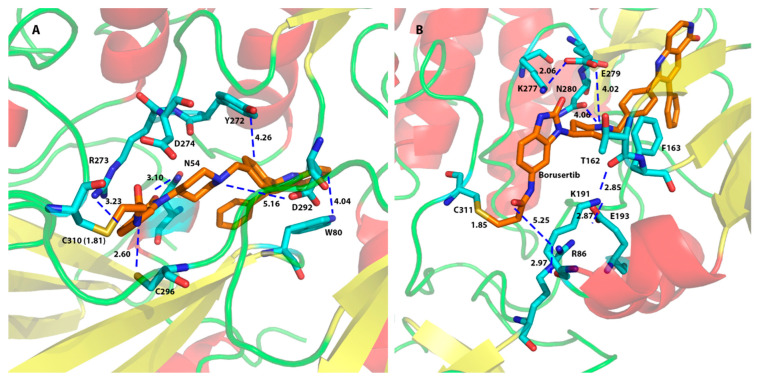
AKT1 (6S9W)/borussertib (**6**) interactions (**A**), and AKT2 (3D0E_N)/borussertib (**6**) interactions (**B**). A covalent bond was formed between CYS310 of AKT1 and the β-carbon of the original α,β-unsaturated amide bond and between CYS311 of AKT2 and borussertib (**6**). Distances between binding residues and borussertib were provided in Å. The α-helices were highlighted in red, and the β-sheets were colored in yellow. The ligand was presented in orange atoms and the protein side chain residues in cyan.

**Figure 4:**
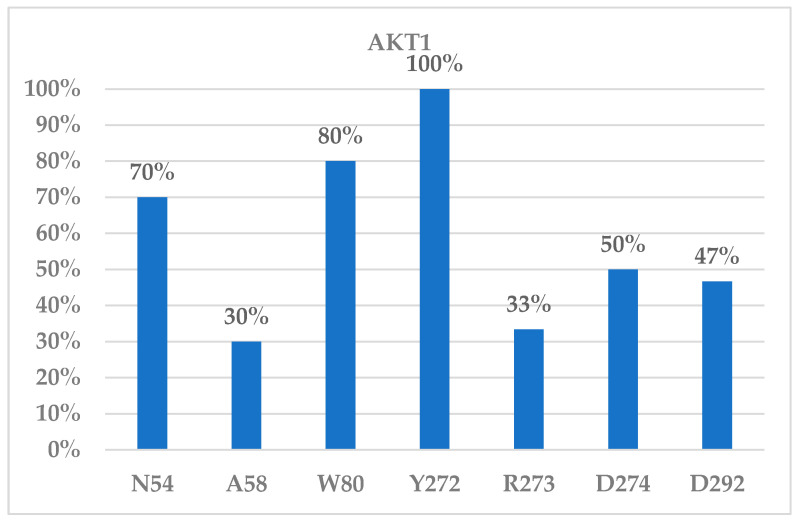
Interacting residues of AKT1 inhibitors.

We investigated the protein–ligand interactions for the top poses yielded for the AKT1 (6S9W) model from the CovDock program and found that residues Asn54, Trp80, Tyr272, Asp274, and Asp292 were the main residues for ligand binding to AKT1. Figure 3A showed that a covalent bond was formed between CYS310 of AKT1 (6S9W model) and the β-carbon of the original α,β-unsaturated amide bond of borussertib (**6**) at a distance between the carbon and the sulfur atoms of 1.81 Å. The interacting residues showed that Asp292 formed an electrostatic interaction with the protonated aliphatic amine (at a distance of 5.16 Å). Trp80 provided an aromatic–aromatic interaction with the aromatic ring of borussertib (**6**) at a distance of 4.04 Å, whereas Asn54 formed an H-bond with the carbonyl oxygen of borussertib (distance between N and O: 3.10 Å). Borussertib interacted with Tyr272 forms through an aromatic–aromatic interaction (distance at 4.26 Å) and with Arg273 via a charge–aromatic ring interaction (distance at 3.23 Å) (Figure 3A). These interactions were observed in most ligands binding to AKT1 (Figure 4).

To visualize the importance of the binding residues that interact with ligands, we counted the number of occurrences of such residues interacting with the reported bound ligands (Figure 4). The binding frequency in Figure 4 showed that Asn54, Trp80, Tyr272, Asp274, and Asp292 of the AKT1 may play an important role in ligand binding. Residues Trp80 and Tyr272 were reported to interact with the allosteric binder ARQ-092 (miransertib). The IC_50_ of ARQ-092 was 5.0, 4.5, and 16 nM for AKT1, AKT2, and AKT3, respectively [44]. However, there was no selectivity for ARQ-092 between the inhibitions of AKT1 and AKT2. An ARQ-092-based proteolysis targeting chimera (PROTAC) degrader was reported with a DC_50_ of 23 nM [45]. The interacting residues for the AKT1 ligand binding are listed in Table S2 (Supplemental Information).

**Figure 5:**
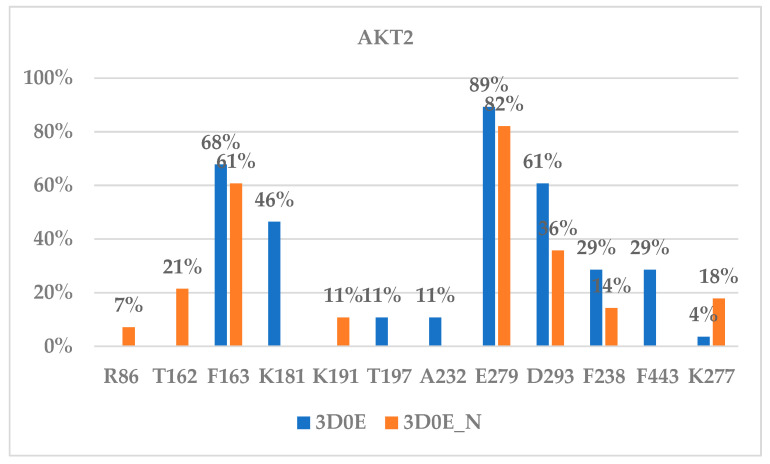
Interacting residues of AKT2 inhibitors.

For the AKT2 ligand binding, Figure 3B showed a covalent bond being formed between Cys311 of AKT2 and borussertib (6) at a distance between the carbon and the sulfur atom of 1.85 Å. Residues Glu279 and Phe163 interacted with most ligands in both the 3D0E and the 3D0E_N models (Figure 5). Glu279 not only provided structural support by forming a charged bridge with Lys277 but also formed an electrostatic interaction with the +1 charged amino group of borussertib (**6**) at a distance of 4.02 Å. Phe163 provided aromatic–aromatic interactions with borussertib (Figure 3B). The corresponding residues of Glu279 and Phe163 in AKT1 and AKT3 are Glu278 (AKT1)/Glu275 (AKT3) and Phe161 (AKT1)/Phe159 (AKT3), respectively (Table 1). The corresponding residues were not observed in ligand binding to the AKT1 and AKT3 in the compounds in this study. Thus, residues Glu279 and Phe163 may play a role in AKT2-specific binding. Asp293 and Lys181 were identified as important for ligand binding in the 3D0E model.

We also enumerated the interacting residues for AKT2 binding to both the 3D0E and 3D0E_N models, and the interacting residues are provided in Table S3 (Supplemental Information) and Figure 5. Table S3 and Figure 5 showed that the majority of residues participating in ligand binding to the AKT2 were in the catalytic domain (residues between 156 and 435). The only two residues in the PH domain that provided interactions with ligand binding were Arg15 and Arg86 observed in the 3D0E_N model (Table S3), and they were interacting with a very small number of ligands. This observation helps to explain why the 3D0E and 3D0E_N models yielded very similar docking scores based on the CovDock method and very similar free energies calculated from the MT method. This suggests that the N-terminal PH domain overall has a minimal effect on ligand binding to the AKT2, though the PH domain residues may participate in one or two ligand interactions.

**Figure 6:**
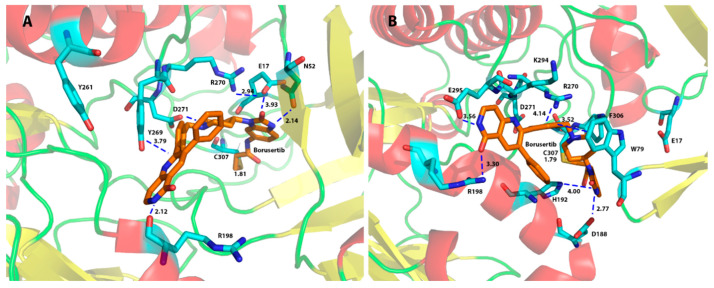
AKT3/borussertib (**6**) interactions from the HM model (**A**) and the AF model (**B**). A covalent bond was formed between CYS307 of AKT3 and the β-carbon of the original α,β-unsaturated amide bond. Distances between binding residues and borussertib were provided in Å. The α-helices were highlighted in red, and the β-sheets were colored in yellow. The ligand was presented in orange atoms and the protein side chain residues in cyan.

The overexpression of AKT3 but not AKT1/2 has been observed in non-small-cell lung cancer (NSCLC) patients who developed resistance against osimertinib [46]. Figure 6 showed AKT3/borussertib (**6**) interactions with the homology model (HM, Figure 6A) and the AlphaFold (AF) model. Both models yielded Asp271 as an important residue for borussertib binding (**6**) via the electrostatic interaction with the +1 charged amino group. Similarly, Arg198 of AKT3 formed an H-bond with the amide bond of **6**. Other residues important for AKT3 ligand binding were given in Table S4 and Figure S4 (Supplemental Information), which included Trp79, Glu295, Phe306, His192, and Glu17 (Figure S4 and Figure 6). The AF model showed that Trp79 formed hydrophobic interactions with borussertib (**6**) and that Asp188 formed H-bonds with the amide bond of **6**.

### 2.4. Binding Selectivity and Drug Design Perspectives

Our docking studies suggest that residues Asn54, Trp80, Tyr272, Asp274, and Asp292 of AKT1 are the main residues for ligand binding to AKT1, residues Glu279 and Phe163 of AKT2 may be responsible for the AKT2-specific binding, and Trp79, Glu295, and Phe306 of AKT3 are the key residues for AKT3 binding. For the Asn54, Trp80, Tyr272, Asp274, and Asp292 of AKT1, the corresponding residues in AKT2 and AKT3 are Asn54 (AKT2)/Asn53 (AKT3), Trp80 (AKT2)/Trp79 (AKT3), Asp275 (AKT2)/Asp271(AKT3), and Asp293 (AKT2)/Asp289(AKT3). Since the Trp79 of AKT3 was the most frequently interacting residue and Asp271 was found to interact with borussertib (Figure 6A) and the Trp80 and Asp274 of AKT1 were important for many ligands’ binding to AKT1, targeting these two residues would yield low selectivity between AKT1 and AKT3, which can be supported by recent publications. The selectivity of NTQ1062 was greater between AKT1 and AKT2 than between AKT1 and AKT3. The IC_50_ of NTQ1062 for AKT1/2/3 was 1.6/24/0.3, respectively [47]. The important residues for AKT2 ligand binding were found to be Glu279 and Phe163, and the corresponding residues in AKT1 and AKT3 were not among the important binding residues. This helps to explain the weaker binding affinity of NTQ1062 toward AKT2 [47].

It needs to be pointed out that borussertib and all other ligands in this study were able to form a covalent bond with Cys310 of AKT1, Cys311 of AKT2, or Cys307 of AKT3. There would be a liability for point mutation-derived resistance if this cysteine was mutated. It is reported that AKT1 mutants C296S and C310S reduced the IC_50_s of borussertib from 0.2 nM to 58 nM, a 290-fold reduction [19].

To overcome the drug resistance to the α,β-unsaturated amide observed in the borussertib, introduction of an aldehyde function group to ARQ-092 (miransertib) would allow the formation of an imine bond with a Lys17 side chain of AKT1, and thus, this modified compound would overcome the drug resistance of the AKT1 E17K mutant, which was identified in 6–8% of breast cancer, 2–6% of colorectal cancers, and 6% of meningiomas [48].

Another approach to overcoming the drug resistance issue is to use the proteolysis-targeting chimeras (PROTACs) technique to develop PROTAC drugs targeting AKT1. A PROTAC-based molecule was developed for the allosteric inhibitor ARQ-092 [45]. PROTAC-based molecules were also reported for the ATP-binding AKT inhibitors capivasertib [49] and ipatasertib [50]. These PROTAC degraders were reported to have impressive degradation concentrations of 50% (DC_50_) in various cancer cells.

## 3. Materials and Methods

### 3.1. Multiple Sequence Alignment

Protein sequences of AKT1 (6S9W) [24], AKT2 (3D0E) [26], and AKT3 (GenBank number: AAD24196.1) were aligned using the server Clustal Omega (https://www.ebi.ac.uk/jdispatcher/msa/clustalo/) (accessed on 5 June 2023) [28]. Residues responsible for ligand binding to different AKT subtypes were provided in Figure S1 (Supplemental Information).

### 3.2. Preparation of Protein Structures

AKT1 model protein 6S9W and AKT2 model protein 3D0E were downloaded from the RCSB Protein Data Bank (www.rcsb.org) and imported to the MOE program [29]. The AKT1 model protein 6S9W started with Asp3 and showed two gaps: the first gap was a large gap from Gln113 to Pro141 and the second was a two-residue small gap between Asp302 and Gly303. The small gap between Asp302 and Gly303 was easily fixed using the MOE Protein Preparation Module (MOE Version: 2022.02). The large 29-residue gap between Gln113 and Pro141 was considered to have a minimum effect on ligand binding during the ligand docking process and thus was not fixed due to the far distance to the active site ligand (19.15 Å between the ligand and residue Lys112). However, to evaluate whether the missing loop would have a positive effect on the ligand binding, we made a model protein by inserting back the large missing loop of Gly113 and Pro141. The MOE Loop Modeler was not able to find a suitable loop structure from the existing database and thus the MOE program made a list of loop structures using the de novo method. The loop structure that fitted the existing structure was adopted. We called this AKT1 model protein 6S9W_L.

For the AKT2 model protein 3D0E, the missing residues Arg455-Asp465 at the C-terminal were far from the binding pocket and thus were deleted. The X-ray structure of 3D0E started from Lys146, which suggested that this structure was missing the N-terminal pleckstrin homology (PH) domain. There were no other gaps between residues Lys146 and Asp454. We refer to this original AKT2 with residues between Lys146 and Asp454 as 3D0E. To evaluate the effect of the PH domain on ligand binding to the AKT2 protein, we made a model protein by attaching the PH domain residues to the model protein 3D0E. To properly incorporate the PH domain to 3D0E, we used the solution structure of the PH domain of AKT2 (PDB id: 1P6S) [34] and the crystal structure of AKT2 (PDB id: 8Q61) [51]. Structural alignment between 1P6S and 8Q61 was carried out in the MOE program to align the PH domain between 1P6S and 8Q61. We also aligned the catalytic domain between 3D0E and 8Q61. The structure 8Q61 was not used in our study because of too many gaps in this structure, and thus, 8Q61 was used as a reference structure to bring 1P6S and 3D0E together. As in AKT1, there was a large gap between the PH domain of 1P6S (which ended with Lys111) and the catalytic domain of 3D0E (starting with Lys146). The coordinates for residues between 112 and 145 were also missing in 8Q61. Thus, we used the MOE Loop Modeler to build this loop structure of AKT2 between Gln112 and Ala145 using the de novo method. We called this model protein 3D0E_N. After model proteins 6S9W, 6S9W_L, 3D0E, and 3D0E_N were prepared, they were subject to the assignment of proper protonation states of residues under pH 7. The ligand structure of borussertib in 6S9W was adopted in model proteins 6S9W_L, 3D0E, and 3D0E_N to enable covalent binding to the respective cysteine of the model proteins. The whole system was subject to minimization first on the side chain only, followed by full optimization using the AMBER14:EHT force field in the MOE program with a gradient of 0.05 RMS kcal/mol/Å^2^. The optimized model proteins were then imported to the Maestro program in the Schrödinger software suite (version: Schrödinger2022-1) [43] for docking study. Prior to the docking study, we prepared the model proteins using the Protein Preparation Wizard in the Schrödinger software to maximize the H-bond interactions and to properly protonate side chain residues under pH 7. The protein was first minimized by limiting the movement of the backbone atoms and then by full optimization using the MacroModel module and the OPLS3e force field. The covalent docking was carried out using the Schrödinger’s CovDock program.

### 3.3. Preparation of a Homology Model for AKT3

There was no reported crystal structure of the catalytic domain of human AKT3 protein, though the PH domain of AKT3 was reported (PDB id: 2X18). Thus, a homology model of human AKT3 was built using the protein primary sequence from the NCBI with sequence ID AAD24196.1 (https://www.ncbi.nlm.nih.gov/protein/AAD24196.1) (accessed on 5 June 2023) [27]. We used the human AKT1 (PDB ID: 6S9W [25]) structure as a template to build the AKT3 HM model. The homology model (HM) of the AKT3 was built using the MOE homology modeling module using default settings. The homology model was minimized first with the side chain atoms and then all atoms were minimized using the AMBER14:EHT force field in the MOE program with a gradient of 0.05 RMS kcal/mol/Å^2^. The alignment of the AKT1 and AKT3_HM model was carried out, and the ligand borussertib of the AKT1 (6S9W) was adopted as the AKT3 ligand to define the covalently binding pocket for the CovDock program by connecting the β-carbon (defined in Figure 1 and Figure 2) and the sulfur atom of CYS307 of AKT3. The AKT3_HM model protein was then prepared using the same procedures and settings as model proteins 6S9W and 3D0E. We also built an AKT3 model (AKT3_AF) using the full-length AKT3 structure provided by the AlphaFold (https://alphafold.ebi.ac.uk/entry/Q9Y243) (accessed on 7 January 2024) [30,31]. We adopted the borussertib in 6S9W of the AKT1 to the AKT3_AF to define the binding ligand for docking studies by aligning these two proteins together. This AKT3_AF then was prepared using the same procedures and same settings as model proteins 6S9W, 6S9W_L, 3D0E, 3D0E_N, and AKT3_HM. After minimization, both AKT3_HM and AKT3_AF were structurally evaluated using the ERRAT and ProCheck programs in the UCLA SAVES v6.0 program [32].

### 3.4. Preparation of Ligand Structures

Thirty ligand structures were taken from Quambusch et al.’s published papers [25,33] and were built using the MOE program [29] with the bound ligand L1Z in 6S9W as a template. Only molecules with well-defined IC_50_ activities for AKT1, AKT2, and AKT3 were included in the discussion. The energy minimization was carried out for all ligands using the MMFF94 force field in the MOE software. These optimized ligands were then transferred to the Maestro program in the Schrödinger suite. The protonation state was assigned to ligands using the EPik program under pH 7.0, and the minimization of ligands was carried out using the MacroModel program with the OPLS3e force field with default settings in the Maestro program [43].

### 3.5. Schrödinger Covalent Docking

To study the effect of the covalent docking, thirty AKT inhibitors were docked to AKT1 (6S9W and 6S9W_L), AKT2 (3D0E and 3D0E_N), and AKT3 (the HM and AF models) proteins using the CovDock program [43] in the Schrödinger software suite using the Michael addition as the reaction type, and the scoring function was set to Virtual Screening. The best docking score was reported. All other parameters were used as defaults.

### 3.6. The Calculation of MT-Based Free Energy

To calculate the Movable Type (MT)-based free energy, coordinates for the protein and ligand are required. Please note that the MT method is not a docking program. It is a method to calculate the free energy of binding. In this study, we used the docked pose generated from the CovDock program. The CovDock reported a protein–ligand complex in a covalently bound manner. To obtain the coordinates for the protein and for the ligand for the MT calculations, we disconnected the covalent C-S bond between borussertib and the cysteine. After disconnecting, the protein was saved in the pdb format. The ligand was modified back to the α,β-unsaturated amide and was saved in the mol2 format, that is, the format the MT program takes as inputs for proteins and ligands. The saved protein and the ligand files were then read by the MT program script, a program developed by Prof. Kenneth Merz Jr. at Michigan State University. The output data of the MT-based method are the absolute free energies of binding in kcal/mol. The reliability of this method has been well documented [35,36,37,39]. Interested readers are referred to the above original papers for more information on the MT method.

### 3.7. Protein–Ligand Interactions and Binding Affinity

We used the docking scores, the CovDock scores (kcal/mol), to estimate the binding affinity of the protein–ligand complexes. The main interacting residues of AKT1, AKT2, and AKT3 were identified to account for hydrogen bonds, electrostatic interactions, and/or aromatic interactions with ligands. The frequencies of the interacting residues of each subtype were reported to show the importance of the interacting residues.

## 4. Conclusions

Many AKT1 inhibitors have been developed for potential anticancer treatment. However, many competitive ATP-binding inhibitors show little selectivity across three subtypes of AKTs. The observed severe adverse events for pan-AKT inhibitors were rash and diarrhea and sometimes hyperlipidemia. The rash was found to be caused by the inhibition of AKT2. It was reported that selective AKT inhibitors that inhibit AKT1 and spare AKT2 showed low cutaneous toxicity. For instance, Hu791 with IC_50s_ for AKT1 and AKT2 of 4.0 and 97.5 nM, respectively was AKT1-selective and thus exhibited less rash. Thus, by selectively inhibiting AKT1 and sparing AKT2 activity, one can develop a molecule with reduced occurrence of rash [23].

Designing an allosteric inhibitor rather than an ATP-based active site inhibitor can minimize the toxicity of inhibiting the ubiquitous ATP binding site. Thus, selective inhibition of allosteric binders that can selectively bind to AKT1 but not AKT2 has the potential to minimize the side effects.

Our data have shown that, although on the sequence level, the binding pockets are highly conserved across AKT1, AKT2, and AKT3, the type of binding residues was not the same. For instance, AKT1 binding may require residues Asn54, Trp80, Tyr272, Asp274, and Asp292, whereas AKT2 binding would expect residues Phe163 and Glu279, and AKT3 binding would favor residues Glu17, Trp79, Phe306, and Glu295. The preference to different types of residues between different subtypes would suggest that ligands with an amino group (RNH_2_) would favor AKT2 binding due to favorable electrostatic interactions with the Glu279 and Asp293 of AKT2; and that an amino group a certain distance away from the aromatic rings may allow it to form interactions withAsp274 and Asp292 as well as with aromatic Trp80 and Tyr272 of AKT1. Nevertheless, it remains challenging to develop subtype selective AKT inhibitors that can exhibit strong binding to AKT1/3 but not to AKT2. Sometimes, it turned out that the inhibition of AKT2 may be the strongest among the three subtypes. For instance, the IC_50_ values of ALM301 against the AKT1, AKT2 and AKT3 were 0.13, 0.09 and 2.75 µM, respectively [51].

## Figures and Tables

**Table 1:** Residues frequently involved in ligand binding to different subtypes of AKT proteins (AKT1: 6S9W; AKT2: 3D0E; AKT3, AAD24196.1, HM/AF). Residues in bold indicate that the residue was involved in ligand binding for individual subtypes.

Subtypes	Residues												
**6S9W/AKT1**	N54	W80	T160	F161	K179	R200	Y272	R273	D274	E278	D292	E298	C310
**3D0E/AKT2**	N54	W80	**T162**	**F163**	**K181**	**F238**	Y273	R274	D275	**E279**	**D293**	E299	**C311**
**HM/AF/AKT3**	N53	**W79**	T158	F159	K177	R198	Y269	R270	D271	E275	D289	**E295**	**C307**

**Table 2:** The CovDock scores (kcal/mol) from the CovDock method (6S9W and 6S9W_L models), and the MT-based free energy (ΔG, kcal/mol) based on the K_i_ (nM) values of the tested compounds and the respective absolute errors (ΔΔGs).

Compd.	K_i_ (nM)	∆Gexp	6S9W	ΔΔG (6S9W)	6S9W_L	ΔΔG (6S9W_L)	MT	∆∆G (MT)
**6**	2	−11.87	−12.94	1.07	−14.695	2.83	−14.62	2.75
**7**	71	−9.75	−11.88	2.13	−14.31	4.56	−12.75	3.00
**8**	1243	−8.06	−13.85	5.80	−14.08	6.02	−14.24	6.18
**10**	59	−9.86	−11.71	1.85	−14.58	4.72	−13.10	3.24
**11**	1432	−7.97	−12.75	4.78	−14.58	6.61	−14.88	6.91
**13**	6	−11.22	−12.03	0.81	−15.56	4.35	−11.71	0.50
**15**	9	−10.98	−12.49	1.51	−13.88	2.90	−12.34	1.36
**17**	8	−11.05	−12.28	1.23	−9.99	1.06	−15.22	4.17
**22**	6	−11.22	−12.65	1.43	−7.65	3.56	−16.64	5.43
**27**	9	−10.98	−12.11	1.13	−9.13	1.85	−16.11	5.14
mean				2.18		3.85		3.87
stdev				1.70		1.75		2.08

**Table 3:** The CovDock scores (kcal/mol) from the CovDock method (3D0E and 3D0E_N models), and the respective MT-based free energies (ΔG, kcal/mol) based on the IC_50_ (nM) values of the tested compounds and the respective absolute errors (ΔΔGs).

Compd.	IC_50_ (nM)	∆G_exp_	3D0E	∆∆G (3D0E)	3D0E_N	∆∆G (3D0E_N)	MT (3D0E)	ΔΔG (MT, 3D0E)	MT (3D0E_N)	ΔΔG (MT, 3D0E_N)
**6**	14	−10.71	−10.49	0.22	−9.00	1.71	−8.94	1.77	−11.19	0.47
**7**	1569	−7.92	−8.60	0.68	−8.05	0.13	−9.96	2.04	−9.75	1.83
**10**	13,030	−6.66	−8.37	1.71	−8.22	1.55	−8.84	2.17	−10.27	3.60
**11**	119	−9.45	−9.66	0.21	−8.38	1.07	−13.38	3.93	−11.73	2.28
**12**	5033	−7.23	−7.14	0.09	−6.39	0.84	−11.16	3.93	−12.10	4.87
**13**	599	−8.49	−9.12	0.63	−9.17	0.69	−12.61	4.12	−11.09	2.60
**14**	94	−9.59	−6.31	3.28	−7.53	2.06	−11.21	1.63	−12.12	2.54
**15**	76	−9.71	−9.09	0.62	−9.89	0.18	−11.46	1.75	−11.58	1.87
**16**	251	−9.00	−10.97	1.96	−11.53	2.53	−9.43	0.43	−11.53	2.53
**17**	53	−9.92	−7.75	2.17	−7.60	2.32	−12.27	2.35	−10.61	0.69
**18**	23	−10.42	−10.31	0.11	−9.66	0.76	−9.61	0.81	−10.92	0.50
**19**	1400	−7.99	−7.77	0.21	−8.33	0.34	−10.67	2.69	−10.87	2.89
**20**	682	−8.41	−8.21	0.20	−8.65	0.24	−11.41	3.00	−11.30	2.89
**21**	133	−9.38	−8.80	0.58	−7.75	1.63	−12.29	2.91	−11.88	2.50
**22**	39	−10.11	−8.84	1.26	−8.50	1.61	−10.24	0.14	−12.29	2.18
**23**	511	−8.58	−4.57	4.01	−9.37	0.78	−9.58	1.00	−13.67	5.09
**24**	150	−9.31	−9.77	0.47	−8.38	0.93	−11.42	2.11	−10.10	0.79
**25**	175	−9.22	−8.29	0.93	−7.30	1.92	−10.73	1.51	−11.30	2.08
**26**	99	−9.55	−8.85	0.71	−10.22	0.66	−10.76	1.21	−10.87	1.31
**27**	55	−9.90	−8.75	1.15	−8.32	1.58	−10.80	0.90	−12.09	2.19
**28**	151	−9.30	−8.24	1.06	−8.31	0.99	−10.88	1.58	−12.35	3.05
**29**	961	−8.21	−11.45	3.24	−9.84	1.63	−13.52	5.31	−11.13	2.92
**30**	248	−9.01	−6.92	2.09	−8.44	0.57	−14.50	5.49	−10.85	1.84
**31**	116	−9.46	−9.54	0.08	−8.20	1.26	−12.61	3.15	−9.90	0.44
**32**	108	−9.50	−9.10	0.41	−7.47	2.03	−13.04	3.53	−10.14	0.63
**33**	479	−8.62	−9.01	0.39	−9.09	0.47	−12.42	3.80	−13.29	4.67
**34**	2068	−7.75	−7.61	0.14	−8.16	0.41	−9.65	1.90	−11.12	3.36
**35**	4214	−7.33	−7.24	0.09	−9.16	1.83	−8.18	0.85	−11.43	4.10
mean				1.03		1.17		2.36		2.38
stdev				1.08		0.69		1.40		1.32

**Table 4:** The CovDock scores (kcal/mol) from the CovDock method (3D0E and 3D0E_N models), and the respective MT-based free energies (ΔG, kcal/mol) based on the K_i_ (nM) values of the tested compounds and the respective absolute errors (ΔΔGs).

Compd.	K_i_ (nM)	∆G_exp_	3D0E	∆∆G (3D0E)	3D0E_N	∆∆G (3D0E_N)	MT (3D0E)	ΔΔG (MT, 3D0E)	MT (3D0E_N)	ΔΔG (MT, 3D0E_N)
**6**	30	−10.26	−10.49	0.23	−9.00	1.26	−8.94	1.32	−11.19	0.92
**11**	57	−9.88	−9.66	0.22	−8.38	1.50	−13.38	3.49	−11.73	1.85
**14**	4	−11.46	−6.31	5.15	−7.53	3.93	−11.21	0.24	−12.12	0.67
**15**	11	−10.86	−9.09	1.77	−9.89	0.97	−11.46	0.61	−11.58	0.72
**17**	40	−10.09	−7.75	2.34	−7.60	2.49	−12.27	2.18	−10.61	0.52
**18**	11	−10.86	−10.31	0.55	−9.66	1.20	−9.61	1.25	−10.92	0.06
**22**	11	−10.86	−8.84	2.01	−8.50	2.36	−10.24	0.61	−12.29	1.43
**25**	95	−9.58	−8.29	1.29	−7.30	2.28	−10.73	1.15	−11.30	1.72
**26**	21	−10.47	−8.85	1.63	−10.22	0.26	−10.76	0.29	−10.87	0.40
**27**	11	−10.86	−8.75	2.11	−8.32	2.53	−10.80	0.05	−12.09	1.23
mean				1.73		1.88		1.12		0.95
stdev				1.43		1.05		1.05		0.59

**Table 5:** The CovDock scores (kcal/mol) for the AKT3 models (the homology model HM, and the AlphaFold model AF), and the MT-based free energy (ΔG, MT, kcal/mol) based on the IC_50_ of the tested compounds and the respective absolute errors (ΔΔGs).

Compd.	IC_50_	∆G_exp_	HM	∆∆G (HM)	AF	∆∆G (AF)	MT (AF)	∆∆G (MT, AF)
**6**	431	−8.68	−10.05	1.36	−5.98	2.70	−9.27	0.59
**11**	16,316	−6.53	−9.14	2.61	−6.22	0.31	−10.36	3.83
**12**	1277	−8.04	−10.74	2.70	−8.02	0.02	−11.39	3.35
**13**	6627	−7.06	−9.52	2.46	−7.21	0.14	−10.00	2.94
**14**	1564	−7.92	−10.25	2.33	−6.80	1.12	−10.43	2.51
**15**	1890	−7.81	−9.86	2.05	−6.56	1.24	−9.20	1.40
**16**	607	−8.48	−10.49	2.01	−6.67	1.81	−10.76	2.28
**17**	3543	−7.44	−10.79	3.36	−6.88	0.56	−11.46	4.03
**18**	356	−8.80	−11.11	2.31	−6.25	2.55	−9.91	1.11
**19**	4356	−7.31	−10.45	3.14	−6.79	0.52	−9.81	2.50
**20**	5339	−7.19	−10.70	3.51	−7.42	0.23	−10.30	3.11
**21**	2108	−7.74	−10.86	3.12	−6.99	0.75	−10.86	3.12
**22**	5266	−7.20	−10.37	3.17	−7.54	0.33	−11.29	4.09
**23**	1509	−7.94	−10.82	2.88	−6.93	1.01	−9.81	1.87
**24**	7509	−6.99	−9.35	2.36	−5.44	1.55	−8.59	1.60
**25**	187	−9.18	−11.17	1.99	−7.15	2.03	−10.66	1.49
**26**	272	−8.96	−10.29	1.33	−6.64	2.31	−10.12	1.17
**27**	3049	−7.52	−10.54	3.01	−6.99	0.53	−10.16	2.63
**28**	6303	−7.09	−11.08	3.99	−6.35	0.74	−8.99	1.89
**30**	6446	−7.08	−9.89	2.81	−5.67	1.41	−7.42	0.34
**31**	3396	−7.46	−9.93	2.47	−5.67	1.80	−6.98	0.48
**32**	5752	−7.15	−10.15	3.00	−6.36	0.79	−9.13	1.98
**35**	8374	−6.93	−10.72	3.80	−6.85	0.07	−9.62	2.69
mean				2.69		1.07		2.22
stdev				0.69		0.82		1.10

**Table 6:** The CovDock scores (kcal/mol) for the AKT3 models (the homology model HM, and the AlphaFold model AF), and the MT-based free energy (ΔG, MT, kcal/mol) based on the K_i_ (nM) values of the tested compounds and the respective absolute errors (ΔΔGs).

Compd.	K_i_	∆G_exp_	HM	∆∆G (HM)	AF	∆∆G (AF)	MT (AF)	∆∆G (MT, AF)
**6**	46.00	−10.01	−10.05	0.04	−5.98	4.03	−9.27	0.74
**18**	29.00	−10.28	−11.11	0.83	−6.25	4.04	−9.91	0.38
**25**	53.00	−9.92	−10.29	0.36	−7.15	2.78	−10.66	0.74
**26**	64.00	−9.81	−10.54	0.72	−6.64	3.17	−10.12	0.31
mean				0.49		3.50		0.54
stdev				0.36		0.63		0.23

## Data Availability

Structural data are available upon request.

## Supplementary Materials

The following supporting information can be downloaded at: https://www.mdpi.com/article/10.3390/molecules29061233/s1. Figure S1. Multiple sequence alignments of three different AKT subtypes. The alignment was generated with the Clustal Omega program. AKT1: 6S9W; AKT2: 3D0E and AKT2_Comb; AKT3: AAD24196.1. Binding residues unique to different subtypes are highlighted. Figure S2. The Ramachandran plot for the AKT3_HM model (A) and the AKT3_AF model (B). Table S1. Docking CovDock scores (Kcal/mol), the MT-based free energy (ΔG, Kcal/mol) and the interacting resdiues for the AKT1 binding (6S9W and 6S9W_L models) based on the IC50 (nM) values of tested compounds. Figure S3. Superposition of AKT1 (red), AKT2 (cyan) and AKT3 (orange). The catalytic domain and the PH domain were highlighted. Table S2. Interacting residues from the CovDock generated poses for the AKT1 (6S9W) models. Table S3. Interacting residues from the CovDock generated poses for the AKT2 (3D0E_N and 3D0E) models. Table S4. Interacting residues from the CovDock generated poses for the AKT3 model (HM model and AF model). Figure S4. Interacting residues of AKT3 inhibitors (the AF model).
