# Supplementary material for: Selectivity Studies and Free Energy Calculations of AKT Inhibitors
Source: Molecules. 2024 Mar 10;29(6):1233. doi: 10.3390/molecules29061233 (PMC10975562; doi:10.3390/molecules29061233)
Supplement: Supplementary file 1 [file molecules-29-01233-s001.zip › molecules-2829274-supplementary.pdf]

Selectivity Studies and Free Energy Calculations of AKT Inhibitors

Haizhen A. Zhong* and David Goodwin

Department of Chemistry, University of Nebraska at Omaha, Omaha, Nebraska 68182, United States
* Correspondence: hzhong@unomaha.edu; Tel.: (1) (402) 554-3145

Figure S1. Multiple sequence alignments of three different AKT subtypes. The alignment was generated with the Clustal Omega program. AKT1: 6S9W; AKT2: 3D0E and AKT2_Comb; AKT3: AAD24196.1. Binding residues unique to different subtypes are highlighted.

Figure S2. The Ramachandran plot for the AKT3_HM model (A) and the AKT3_AF model (B).

Table S1. Docking CovDock scores (Kcal/mol), the MT-based free energy (ΔG, Kcal/mol) and the interacting resdiues for the AKT1 binding (6S9W and 6S9W_L models) based on the IC_{50} (nM) values of tested compounds.

Figure S3. Superposition of AKT1 (red), AKT2 (cyan) and AKT3 (orange). The catalytic domain and the PH domain were highlighted.

Table S2. Interacting residues from the CovDock generated poses for the AKT1 (6S9W) models.

Table S3. Interacting residues from the CovDock generated poses for the AKT2 (3D0E_N and 3D0E) models.

Table S4. Interacting residues from the CovDock generated poses for the AKT3 model (HM model and AF model).

Figure S4. Interacting residues of AKT3 inhibitors (the AF model).

```
CLUSTAL 2.1 multiple sequence alignment

6S9W (AKT1)      GSDVAIVKEGWLHKRGEYIKTWRPRYFLLKNDGTFIGYKERPQDVDQREAPLNNFSVAQC 60
AAD24196.1       MSDVTIVKEGWVQKRGEYIKNWRPRYFLLKTDGSFIGYKEKPQDVDLP-YPLNNFSVAKC 59
3D0E (AKT2)      ------------------------------------------------------------ 0
AKT2_Comb        MNEVSVIKEGWLHKRGEYIKTWRPRYFLLKSDGSFIGYKERPEAPDQTLPPLNNFSVAEC 60
                                  E17 (AKT3)                           N54 (AKT1/2)
                                                                      N53 (AKT3)

6S9W (AKT1)      QLMKTERPRPNTFIIRCLQWTTVIERTFHVETPEEREEWTTAIQTVADGLKKQAAAE--M 118
AAD24196.1       QLMKTERPKPNTFIIRCLQWTTVIERTFHVDTPEEREEWTEAIQAVADRLQRQEE----E 115
3D0E (AKT2)      ------------------------------------------------------------ 0
AKT2_Comb        QLMKTERPRPNTFVIRCLQWTTVIERTFHVDSPDEREEWMRAIQMVANSLKQRAPGEDPM 120
                                   W80 (AKT1)
                                   W79 (AKT3)
                                          R86 (AKT2)
6S9W (AKT1)      DFRSGSP--SDNSGAEEMEVSLAKPKHRVTMNEFEYLKLLGKGTFGKVILVKEKATGRYY 176
AAD24196.1       RMNCSPTSQIDNIGEEEMDA-STTHHKRKTMNDFDYLKLLGKGTFGKVILVREKASGKYY 174
3D0E (AKT2)      ---------------------------KVTMNDFDYLKLLGKGTFGKVILVREKATGRYY 33
AKT2_Comb        DYKCGSPSD--SSTTEEMEVAVSKARAKVTMNDFDYLKLLGKGTFGKVILVREKATGRYY 178
                                            :  ***:*:*****************:***:*:**
                                                        T162, F163 (AKT2)

6S9W (AKT1)      AMKILKKEVIVAKDEVAHTLTENRVLQNSRHPFLTALKYSFQTHDRLCFVMEYANGGELF 236
AAD24196.1       AMKILKKEVIIAKDEVAHTLTESRVLKNTRHPFLTSLKYSFQTKDRLCFVMEYVNGGELF 234
3D0E (AKT2)      AMKILRKEVIIAKDEVAHTVTESRVLQNTRHPFLTALKYAFQTHDRLCFVMEYANGGELF 93
AKT2_Comb        AMKILRKEVIIAKDEVAHTVTESRVLQNTRHPFLTALKYAFQTHDRLCFVMEYANGGELF 238
                 *****:****:*******:**.***:*:******:***:***:*********.******
                          D188, H192,   R198 (AKT3)
                   K181             K191 E193 (AKT2)
6S9W (AKT1)      FHLSRERVFSEDRARFYGAEIVSALDYLHSEKNVVYRDLKLENLMLDKDGHIKITDFGLC 296
AAD24196.1       FHLSRERVFSEDRTRFYGAEIVSALDYLHSG-KIVYRDLKLENLMLDKDGHIKITDFGLC 293
3D0E (AKT2)      FHLSRERVFTEERARFYGAEIVSALEYLHSR-DVVYRDIKLENLMLDKDGHIKITDFGLC 152
AKT2_Comb        FHLSRERVFTEERARFYGAEIVSALEYLHSR-DVVYRDIKLENLMLDKDGHIKITDFGLC 297
                 *********:*:*:************:****   .:****:*********************
                                 (AKT1)            Y272,R273,D274        D292 C296
                                 (AKT2)                K277, E279,N280    D293
                                 (AKT3)              R270, D271

6S9W (AKT1)      KEGIKDGATMKTFCGTPEYLAPEVLEDNDYGRAVDWWGLGVVMYEMMCGRLPFYNQDHEK 356
AAD24196.1       KEGITDAATMKTFCGTPEYLAPEVLEDNDYGRAVDWWGLGVVMYEMMCGRLPFYNQDHEK 353
3D0E (AKT2)      KEGISDGATMKTFCGTPEYLAPEVLEDNDYGRAVDWWGLGVVMYEMMCGRLPFYNQDHER 212
AKT2_Comb        KEGISDGATMKTFCGTPEYLAPEVLEDNDYGRAVDWWGLGVVMYEMMCGRLPFYNQDHER 357
                 ****.*.*****************************************************:
                                C310 (AKT1), C311 (AKT2)
                 K294, E295, F306,C307 (AKT3)

6S9W (AKT1)      LFELILMEEIRFPRTLGPEAKSLLSGLLKKDPKQRLGGGSEDAKEIMQHRFFAGIVWQHV 416
AAD24196.1       LFELILMEDIKFPRTLSSDAKSLLSGLLIKDPNKRLGGGPDDAKEIMRHSFFSGVNWQDV 413
3D0E (AKT2)      LFELILMEEIRFPRTLSPEAKSLLAGLLKKDPKQRLGGGPSDAKEVMEHRFFLSINWQDV 272
AKT2_Comb        LFELILMEEIRFPRTLSPEAKSLLAGLLKKDPKQRLGGGPSDAKEVMEHRFFLSINWQDV 417
                 ********:*:*****.  :*****:***  ***::*****  .****:*.*  **  .:  **.*
```

Reported sequence identity between sequences:

```
        1: 6S9W (AKT1) 100.00   82.31   88.74   83.30
        2: AAD24196.1   82.31  100.00   83.28   78.79
        3: 3D0E (AKT2)  88.74   83.28  100.00  100.00
        4: AKT2_Comb    83.30   78.79  100.00  100.00
```

Figure S1. Multiple sequence alignments of three different AKT subtypes. The alignment was generated with the Clustal Omega program. AKT1: 6S9W; AKT2: 3D0E and AKT2_Comb; AKT3: AAD24196.1. Binding residues unique to different subtypes are highlighted.

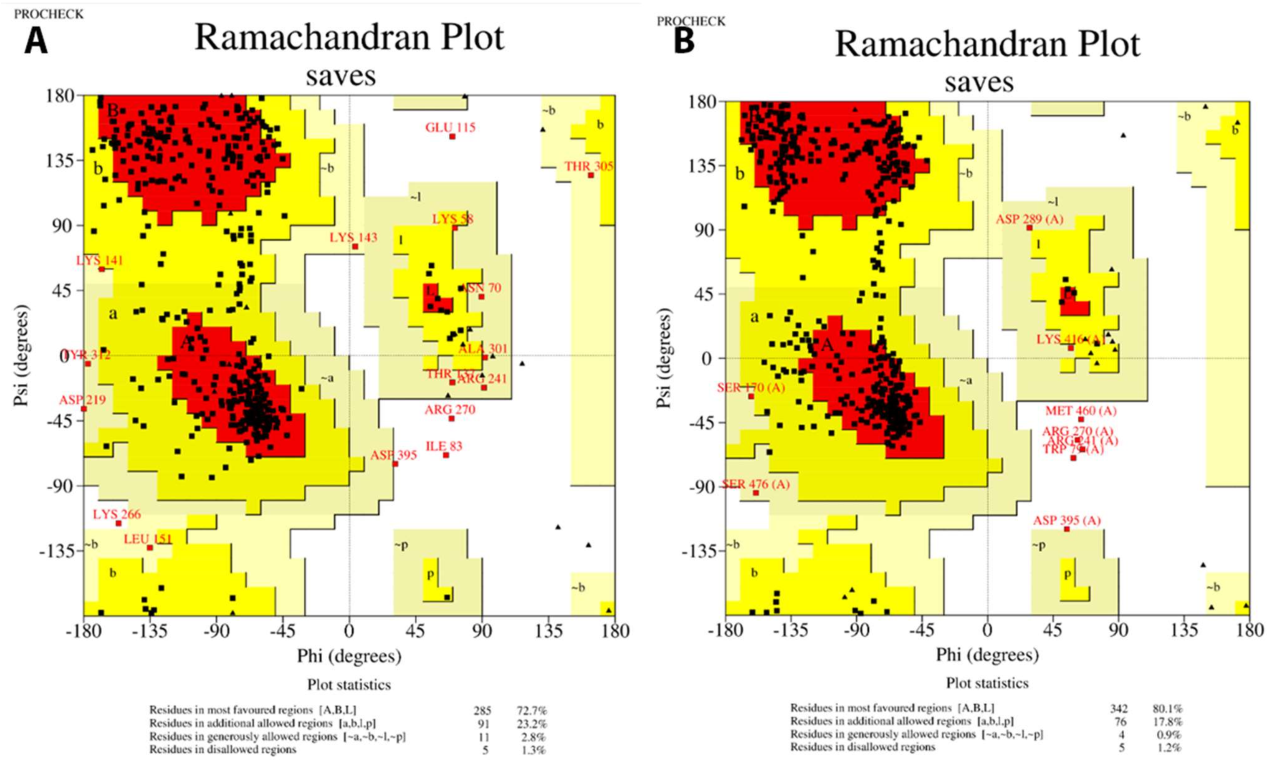

Figure S2. The Ramachandran plot for the AKT3_HM model (A) and the AKT3_AF model (B).

Table S1. Docking CovDock scores (Kcal/mol), the MT-based free energy (ΔG, Kcal/mol) and the interacting resdiues for the AKT1 binding (6S9W and 6S9W_L models) based on the IC_{50} (nM) values of tested compounds.

Compd.	IC_{50} (nM)	ΔG_{exp}	6S9W	ΔΔG (6S9W)	6S9W_L	ΔΔG (6S9W_L)	MT1	ΔΔG (MT, 6S9W)
**6**	1	-12.28	-12.94	0.66	-14.695	2.42	-14.62	2.34
**7**	38	-10.12	-11.88	1.76	-14.31	4.19	-12.75	2.63
**8**	490	-8.61	-13.85	5.25	-14.08	5.47	-14.24	5.63
**9**	381	-8.76	-14.12	5.36	-13.84	5.09	-15.66	6.90
**10**	39	-10.11	-11.71	1.61	-14.58	4.48	-13.10	3.00
**11**	1140	-8.11	-12.75	4.64	-14.58	6.48	-14.88	6.77
**12**	813	-8.31	-11.64	3.33	NA		-16.61	8.30
**13**	53	-9.92	-12.03	2.10	-15.56	5.64	-11.71	1.79
**14**	1716	-7.86	-12.76	4.89	-12.11	4.24	-15.00	7.14
**15**	643	-8.45	-12.49	4.04	-13.88	5.43	-12.34	3.89
**16**	1281	-8.04	-12.86	4.82	-12.19	4.15	-14.62	6.58
**17**	98	-9.56	-12.28	2.72	-9.99	0.43	-15.22	5.65
**18**	527	-8.56	-13.18	4.62	-14.18	5.61	-16.32	7.76

**19**	604	-8.48	-13.17	4.68	-14.54	6.05	-15.41	6.93
**20**	9484	-6.85	-12.90	6.05	-12.67	5.82	-15.40	8.55
**21**	2853	-7.56	-12.00	4.44	-13.27	5.70	-17.15	9.59
**22**	91	-9.60	-12.65	3.04	-7.65	1.95	-16.64	7.04
**23**	1797	-7.84	-12.94	5.10	-12.49	4.65	-15.32	7.48
**24**	22	-10.45	-10.82	0.37	-15.47	5.02	-12.63	2.18
**25**	1631	-7.89	-11.02	3.13	-9.47	1.57	-13.81	5.91
**26**	2111	-7.74	-13.07	5.32	-11.81	4.07	-15.81	8.07
**27**	209	-9.11	-12.11	3.00	-9.13	0.02	-16.11	7.00
**28**	130	-9.39	-11.22	1.82	-12.18	2.79	-15.62	6.23
**29**	186	-9.18	-11.71	2.53	-15.91	6.73	-13.39	4.20
**30**	44	-10.04	-11.90	1.87	-15.30	5.27	-14.52	4.48
**31**	35	-10.17	-11.44	1.27	-14.56	4.38	-13.24	3.07
**32**	112	-9.48	-11.66	2.18	-14.38	4.90	-14.34	4.86
**33**	7087	-7.02	-12.55	5.53	-13.78	6.75	-13.67	6.65
**34**	4566	-7.28	-12.15	4.87	-13.94	6.66	-16.56	9.27
**35**	5930	-7.13	-12.92	5.79	-13.30	6.17	-13.98	6.85

mean				3.56		4.56		5.89
stdev				1.66		1.81		2.18

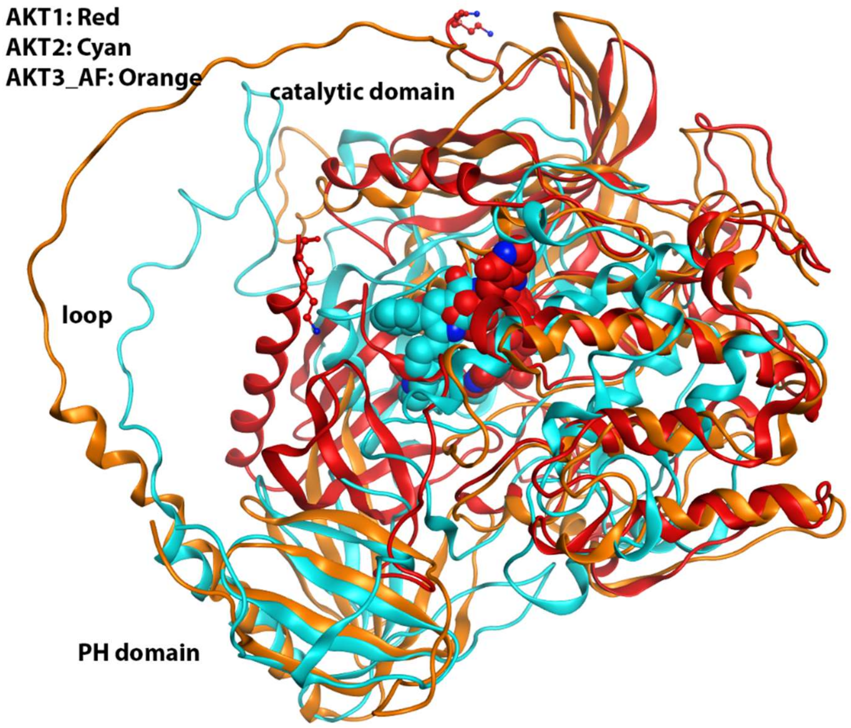

Figure S3. Superposition of AKT1 (red), AKT2 (cyan) and AKT3 (orange) and the catalytic domain and the PH domain of the AKT proteins.

Table S2. Interacting residues from the CovDock generated poses for the AKT1 (6S9W) models.

**Compd.**	**Interacting residues (6S9W)**	**Compd.**	**Interacting residues (6S9W)**
**6**	N54, W80, Y272, D292	**21**	E17, Q79, W80, N204, E267, Y272, D274
**7**	N54, W80, Y263, Y272, D274, D292	**22**	N54, A58, Q59, W80, Y272, D292
**8**	N54, W80, Y272	**23**	E17, Q79, W80, Y272, R273, D274
**9**	N53, N54, W80, Y263, Y272, D292, L295	**24**	N54, W80, Y272, D292
**10**	N54, W80, Y272, D292	**25**	E17, Q79, Y272, R273, D274, E267
**11**	E17, Q79, W80, Y272, R273, D274, C310	**26**	N54, W80, Y272, D274, D292
**12**	E17, Q79, Y272, R273, D274,K297, C310	**27**	N54, W80, Y272, D292, L295, K297
**13**	N54, W80, Y272, D292	**28**	N54, W80, Y272, R273, C310
**14**	E17, Q79, Y272, R273, D274, C310	**29**	N54, W80, Y272, D292
**15**	E17, N54, Q79, Y272, R273, D274	**30**	N54, W80, Y272, D292
**16**	E17, Q79, Y263, Y272, R273, D274, C310	**31**	N54, W80, Y272,D274
**17**	N54, W80, Y272, D274, D292	**32**	N54, W80, Y272
**18**	N54, W80, E267, Y272	**33**	E17, Q79, Y272, R273, D274
**19**	N54, W80, Y272, D274, D292	**34**	N54, W80, Y272, C310
**20**	E17, A58, W80, R200, E267, Y272, R273, D274, C310	**35**	N54, W80, T82, Y272, D292

Table S3. Interacting residues from the CovDock generated poses for the AKT2 (3D0E_N and 3D0E) models.

Compd.	Interacting residues (3D0E_N)	Interacting residues (3D0E)
6	F163, K191, E279, D293	F163, K181, E230, A232, E279, D293
7	F163, K277, E279, D293	F163, K181, T197, E279, D293
10	E279	F163, K181, E279, D293
11	F163, E279, K277, D293	F163, E279, D293, K181
12	E279	F163, D293
13	E279	F163, K181, E279, D293
14	F163, K277, D293	F163, E279
15	F163, K191, E193, E279	F163, K181, T197, E279, D293
16	E193, A232, E279	F163, K181, T197, A232, E279, D293
17	R15, K277, N280	F238, E279, F443
18	E193, E279, A232	F163, K181, E279, D293
19	F163,T162, E279, F238	F163, E279, D293
20	F163, E230, E279, D293	F163, E279
21	T162, F238, E279	F238, E279, F443
22	T213, E279	F238, E279, F443
23	F163, E230, E279	E193, K277, L278
24	F163, N280, E279, D293	F163, K181, E279, D293
25	F163, T213, F238, E279, K277	F238, E279, D293
26	F163, T162, E279	F238,E279, T313, F443
27	E279	F238, E279, F443
28	R86, F163, D293	F238, E279, F443
29	E279, E193, T213	F163, K181, A232, E279, D293
30	E279, E193	F163, E279, D293, G312
31	F163, T162, D293	F163, K181, A232, D293
32	F163, T162, E279, D293	F163, K181, T197, E279, D293, F443
33	F163, E193, E230, E279	F163, K181, E279, D293
34	F163, T162, F238	F238, E279, F443
35	F163, K191, E236, E279, D293	F163, E279

Note: the reduced number of protein-ligand interactions in the AKT2 model was since ligands with >20,000 nM were removed from further analysis.

Table S4. Interacting residues from the CovDock generated poses for the AKT3 AF model.

Compd.	Interacting residues (AF)	Compd	Interacting residues (AF)

**6**	W79, K294, D188, H192, F306	**22**	W79, F306
**11**	E17, W79	**23**	G308
**12**	E17, W79, E295	**24**	W79, E17
**13**	W79, H192, R198, K294, F306	**25**	E17, T195, E295
**14**	W79, T195, R270, F306	**26**	W79, E295
**15**	E17	**27**	W79, T195, R270, F306, G308
**16**	W79, E295, G308	**28**	NA
**17**	W79, F306	**30**	W79, H192 , R198
**18**	E295	**31**	W79, H192, Q470
**19**	E17, W79, E295	**32**	W79, H192, E295, K294, D188
**20**	E295, G308	**35**	W79, K294, E295, H192, R198, D188, F306
**21**	W79, F306		

Note: the reduced number of protein-ligand interactions in the AKT3 model was since ligands with >20,000 nM were removed from further analysis.

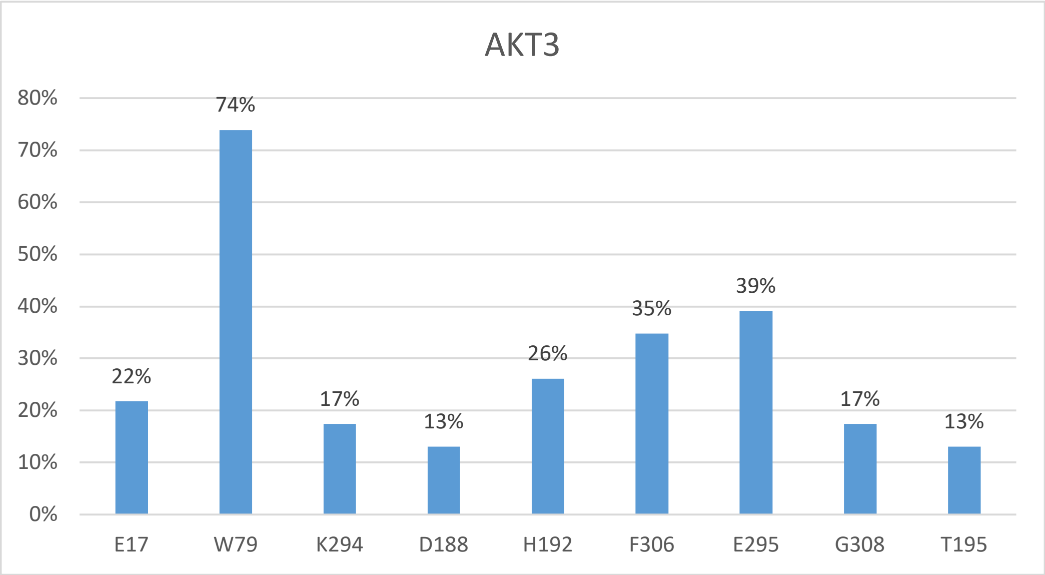

Figure S4. Interacting residues of AKT3 inhibitors (the AF model).
